# Safety Profile and Bleeding Complications of Fondaparinux Versus Enoxaparin in Non‐ST Elevation Acute Coronary Syndrome

**DOI:** 10.7759/cureus.99880

**Published:** 2025-12-22

**Authors:** Md. Mahfuzur Rahman, Farid Uddin Ahmed, Mohammad Ibrahim Chowdhury, Hafsa Noor, Md. Akram Hossain

**Affiliations:** 1 Cardiology, Chittagong Medical College, Chattogram, BGD; 2 Community Medicine, Rangamati Medical College, Rangamati, BGD; 3 General Practice, 250 Bed General Hospital, Noakhali, BGD; 4 Cardiology, Chattogram General Hospital, Chittagong, BGD

**Keywords:** : acute coronary syndrome, bleeding complications, enoxaparin sodium, fondaparinux, non-st segment elevation acute coronary syndrome

## Abstract

Background

Non-ST-elevation acute coronary syndrome (NSTE-ACS) remains a major contributor to morbidity and mortality worldwide. Optimal anticoagulation is critical for preventing complications, but the choice between fondaparinux and enoxaparin remains debated, particularly regarding bleeding and ischemic outcomes. The objective of this study was to compare the safety profile, bleeding complications, ischemic outcomes, mortality, and composite adverse events between fondaparinux and enoxaparin in NSTE-ACS patients in Bangladesh.

Methods

This prospective observational study enrolled 177 NSTE-ACS patients from Noakhali Medical College and Hospital, Bangladesh, between January and December 2022. Patients received either fondaparinux (n=87) or enoxaparin (n=90). Bleeding and ischemic events, mortality, and composite outcomes were assessed at baseline, 3-month, and 6-month follow-ups. Multivariable logistic regression was performed to evaluate predictors of composite outcomes.

Results

Total bleeding incidence was significantly lower in fondaparinux-treated patients (6.90% vs. 23.33%, p=0.002), notably during index hospitalization (4.60% vs. 17.78%, p=0.006). Fondaparinux significantly reduced myocardial infarction (3.45% vs. 14.44%, p=0.016), recurrent ischemia (11.49% vs. 24.44%, p=0.025), and hospitalization due to heart failure (4.60% vs. 14.44%, p=0.026). Mortality was lower with fondaparinux but not statistically significant (13.79% vs. 25.56%, p=0.050). The composite endpoint of mortality and ischemic events was significantly lower in fondaparinux-treated patients (20.69% vs. 40.00%, p=0.005). Multivariable analysis revealed fondaparinux as an independent protective factor against composite outcomes (OR=0.336, 95% CI: 0.166-0.683, p=0.003), with no significant predictive value found for age, sex, hypertension, diabetes, smoking, or time to intervention.

Conclusions

Fondaparinux demonstrates superior safety and effectiveness over enoxaparin in reducing bleeding complications and ischemic events among NSTE-ACS patients. These findings support its preferential use in resource-constrained clinical settings.

## Introduction

Acute coronary syndrome (ACS) remains a major global health burden, with ischemic heart disease (IHD) ranking as the leading cause of death worldwide. In 2019, the Global Burden of Disease study estimated that over 17.9 million deaths were attributed to cardiovascular diseases (CVDs), with a significant proportion resulting from ACS [1]. Among the ACS subtypes, non-ST elevation ACS (NSTE-ACS), comprising unstable angina (UA) and non-ST elevation myocardial infarction (NSTEMI), accounts for over 60% of ACS hospital admissions in high-income countries, and its prevalence is rising in low- and middle-income countries (LMICs), including Bangladesh [2]. The effective management of NSTE-ACS relies on early initiation of antiplatelet therapy, anticoagulation, and, where indicated, an early invasive strategy [3]. Among anticoagulants, low-molecular-weight heparins (LMWHs), particularly enoxaparin, have long been considered the standard therapy; however, concerns regarding bleeding risk have prompted the exploration of alternative agents such as fondaparinux, a selective factor Xa inhibitor [4]. The landmark OASIS-5 trial, which evaluated over 20,000 ACS patients, demonstrated that fondaparinux significantly reduced major bleeding (2.2% vs. 4.1%, p < 0.01) compared to enoxaparin, without compromising ischemic efficacy [4]. Additional studies confirm that fondaparinux use in clinical practice leads to lower rates of major bleeding and improved overall safety, particularly in elderly and high-bleeding-risk populations [5]. However, fondaparinux must be supplemented with unfractionated heparin (UFH) during percutaneous coronary intervention (PCI) to prevent catheter-related thrombosis, a factor that may affect its adoption in real-world settings [6]. Bleeding complications in anticoagulated ACS patients remain a critical concern, as they increase mortality, prolong hospital stays, and significantly impact healthcare costs [7]. Studies have shown that major bleeding not only leads to higher transfusion rates but also results in the early discontinuation of antithrombotic therapy, thereby increasing the risk of recurrent ischemic events [8]. Furthermore, bleeding events have been linked to a threefold increase in mortality among ACS patients [9]. These concerns are particularly relevant in resource-limited settings like Bangladesh, where blood product availability is often constrained, and healthcare systems are not optimally equipped to manage major bleeding [10]. In Bangladesh, the management of ACS presents unique challenges due to limited PCI availability, financial constraints, and inconsistent adherence to guideline-directed anticoagulation strategies [11]. While enoxaparin remains the most frequently used anticoagulant, the real-world use of fondaparinux in Bangladeshi ACS patients remains poorly documented [12]. Despite the demonstrated safety benefits of fondaparinux in large-scale global trials, its cost-effectiveness, accessibility, and real-world bleeding risk in Bangladesh remain uncertain. The OASIS-5 findings, although groundbreaking, were largely conducted in high-income settings, making their direct application to Bangladesh's unique patient demographics, comorbidities, and healthcare infrastructure questionable [9]. Given these gaps, there is an urgent need for region-specific research to evaluate the safety profile and bleeding risks associated with fondaparinux compared to enoxaparin in real-world Bangladeshi NSTE-ACS patients. Understanding local bleeding risks, mortality rates, and cost-effectiveness will be crucial in guiding anticoagulation choices in this setting [13]. This study aimed to fill the knowledge gap by providing observational data on bleeding complications and safety outcomes associated with use of fondaparinux versus enoxaparin in Bangladesh.

## Materials and methods

This prospective observational study was conducted at the Department of Cardiology, Noakhali Medical College & Hospital, Bangladesh, over one year from January to December 2022. The study received ethical clearance from the Institutional Review Board of Noakhali Medical College (approval number 005/2021), and formal permission for data collection was obtained from hospital authorities. Written informed consent was secured from all participants after clearly explaining the study's purpose, procedures, and potential risks.

Consecutively admitted patients diagnosed with NSTE-ACS, including unstable angina and NSTEMI, were considered eligible for inclusion. Patients were excluded if they had contraindications to low-molecular-weight heparin (LMWH), recent hemorrhagic stroke, other indications for long-term anticoagulation, serum creatinine ≥3.0 mg/dL, pregnancy, a diagnosis of ST-elevation myocardial infarction (STEMI), or life-limiting comorbidities with an expected survival of less than six months.

A total of 177 patients met the eligibility criteria and were included in the study, with 87 patients receiving fondaparinux and 90 receiving enoxaparin. Patients in the fondaparinux group received 2.5 mg once daily via subcutaneous injection, typically for an average duration of 6 days or until hospital discharge, whichever came earlier. In the enoxaparin group, patients were administered 1 mg/kg subcutaneously twice daily; however, if their creatinine clearance was below 30 mL/min, the dose was adjusted to 1 mg/kg once daily. Enoxaparin was administered for a minimum of 2 days and up to 8 days or until clinical stabilization was achieved. Both treatment protocols adhered to current international guidelines for the management of NSTE-ACS [14]. All patients received standard adjunct therapies, including dual antiplatelet therapy with aspirin and clopidogrel, beta-blockers, angiotensin-converting enzyme (ACE) inhibitors or angiotensin II receptor blockers (ARBs), and statins, as clinically indicated.

The primary efficacy outcome was the prevention of a composite of death, myocardial infarction, recurrent ischemia, stroke, heart failure, and hospitalization due to heart failure within a six-month follow-up period. The primary safety outcome was the incidence of bleeding, classified according to the Bleeding Academic Research Consortium (BARC) criteria, with types 3 and 5 considered major bleeding and types 1 and 2 considered minor bleeding. Secondary outcomes included individual event rates for ischemic complications and mortality during index hospitalization, 3 months, and 6 months. Patients were closely monitored during hospitalization for immediate adverse events and were followed up monthly for six months post-discharge, either in person or via phone contact.

Data collected included demographic and clinical characteristics, comorbidities, biochemical parameters (e.g., hemoglobin, creatinine), ejection fraction, medication use, and timing of intervention. All data were entered and analyzed using IBM SPSS Statistics (Version 23.0). Descriptive statistics were reported as mean with standard deviation (SD) for continuous variables and frequencies with percentages for categorical variables. For between-group comparisons, the Chi-square test or Fisher’s exact test was used for categorical variables, while the independent t-test was used for continuous variables, depending on data distribution. A p-value of less than 0.05 was considered statistically significant. Additionally, multivariable logistic regression analysis was conducted to identify independent predictors of the composite outcome.

## Results

Baseline demographic and clinical characteristics were well balanced between the two treatment groups (Table 1). The mean age was nearly identical in patients receiving fondaparinux (60.2±8.3 years) and those receiving enoxaparin (60.1±10.7 years), with no significant difference (t=0.70, p=0.928). Similarly, BMI distribution and mean values were comparable across groups (23.1±2.9 vs. 22.8±3.6, t=-1.14, p=0.446). While there was a trend toward a higher proportion of males in the enoxaparin group (83.3% vs. 71.3%), this did not reach statistical significance (χ²=3.03, p=0.055). The prevalence of common cardiovascular risk factors, including hypertension, diabetes, dyslipidemia, smoking, prior CAD, and prior stroke, showed no statistically significant differences (all with p>0.19). Time from symptom onset to intervention was also similar between groups, both in categorical distribution (≤6 hours, 7-12 hours, 19-24 hours) and mean duration (7.26±2.03 vs. 7.31±3.54 hours, t=-1.14, p=0.915). Overall, these findings indicate that the two groups were clinically comparable at baseline, minimizing potential confounding due to imbalances in demographic or comorbidity profiles.

**Table 1:** Baseline characteristics of NSTE-ACS patients treated with fondaparinux versus enoxaparin (N=177) Data are presented as number (percentage) or mean ± standard deviation (SD), as appropriate. Statistical comparisons were performed using chi-square (χ²) tests for categorical variables and independent t-tests for continuous variables. A p-value of <0.05 was considered statistically significant.

Variable	Fondaparinux (n=87)	Enoxaparin (n=90)	Test Statistics	p-value
	n (%)	n (%)		
Age				
<50 years	8(9.20%)	11(12.22%)	χ²=0.45	0.799
50-64 years	46(52.87%)	47(52.22%)		
65+ years	33(37.93%)	32(35.56%)		
Mean±SD	60.23±8.31	60.10±10.71	t=0.70	0.928
BMI				
Underweight (<18.5)	1(1.15%)	4(4.44%)	χ²=0.36	0.143
Normal (18.5-24.9)	66(75.86%)	64(71.11%)		
Overweight (25-29.9)	17(19.54%)	22(24.44%)		
Obese (>=30)	3(3.45%)	0(0.00%)		
Mean±SD	23.14±2.93	22.76±3.63	t=–1.14	0.446
Sex				
Male	62(71.26%)	75(83.33%)	χ²=3.03	0.055
Female	25(28.74%)	15(16.67%)		
Comorbidities and risk factors				
Hypertension	57(65.52%)	54(60.00%)	χ²=0.36	0.448
Smoking	57(65.52%)	67(74.44%)	χ²=1.28	0.195
Dyslipidemia	38(43.68%)	42(46.67%)	χ²=0.00	0.690
Diabetes	37(42.53%)	37(41.11%)	χ²=0.06	0.848
History of CAD	21(24.14%)	22(24.44%)	χ²=0.00	0.962
Stroke	7(8.05%)	7(7.78%)	χ²=0.00	0.947
Time from onset to Intervention				
<=6 hours	30(34.48%)	35(38.89%)	χ²=0.48	0.289
7-12 hours	57(65.52%)	53(58.89%)		
19-24 hours	0(0.00%)	2(2.22%)		
Mean±SD	7.26±2.03 hours	7.31±3.54 hours	t=–1.14	0.915

The incidence of bleeding was consistently higher in patients treated with enoxaparin compared with fondaparinux, with significant differences observed during hospitalization and in cumulative events. During the index hospitalization, bleeding occurred in 17.8% of patients in the enoxaparin group versus only 4.6% in the fondaparinux group, representing a statistically significant difference (χ²=6.41, p=0.006). At 3 months, bleeding was reported in 3.3% of enoxaparin patients compared with 1.2% of fondaparinux patients, though this difference was not significant (p=0.747). A similar nonsignificant trend persisted at 6 months (2.2% vs. 1.2%, p=0.970). However, when all time points were pooled, the cumulative incidence of bleeding was substantially higher in the enoxaparin group (23.3%) compared to the fondaparinux group (6.9%), and this difference was highly significant (χ²=8.02, p=0.002). These findings highlight a clinically meaningful advantage of fondaparinux in terms of reducing bleeding risk (Table 2).

**Table 2:** : Incidence of Bleeding Events Among NSTE-ACS Patients by Treatment Group at different time intervals (N=177) Data are presented as number (percentage). Statistical comparisons were performed using chi-square (χ²) tests for categorical variables and Fisher’s exact tests where appropriate. A p-value of <0.05 was considered statistically significant. c: statistical significance analyzed using chi-square test, f: statistical significance analyzed using Fisher's test.

Bleeding	Fondaparinu (n=87)	Enoxaparin (n=90)	Test Statistics (χ²)	p-value
	n(%)	n(%)		
At the index hospitalization	4(4.60%)	16(17.78%)	6.41	0.006c
at 3 months	1(1.15%)	3(3.33%)	Fisher	0.747f
At 6 months	1(1.15%)	2(2.22%)	Fisher	0.970f
Total Bleeding Incidence	6(6.90%)	21(23.33%)	8.02	0.002c

The occurrence of ischemic complications tended to be more frequent in the enoxaparin group across most time points, with some outcomes reaching statistical significance (Table 3). During hospitalization, recurrent ischemia was observed in 17.8% of enoxaparin patients compared to 9.2% in the fondaparinux group, though this difference narrowly missed statistical significance (χ²=2.10, p=0.095). Heart failure during the same period was slightly higher in enoxaparin patients (23.3% vs. 19.5%), but not statistically significant (χ²=0.19, p=0.539). At 3 months, recurrent ischemia (8.9% vs. 2.3%, p=0.099) and myocardial infarction (4.4% vs. 2.3%, p=0.682) remained more common in the enoxaparin group, although not statistically significant. At 6 months, however, the rate of myocardial infarction was significantly higher with enoxaparin (10.0%) compared to fondaparinux (1.1%) (Fisher’s exact, p=0.008). Other 6-month outcomes, such as recurrent ischemia, stroke, and heart failure, showed higher rates in the enoxaparin group but did not reach statistical significance. When outcomes were pooled across all follow-up points, statistically significant differences were observed: total myocardial infarction (14.4% vs. 3.5%; p = 0.016), total recurrent ischemia (24.4% vs. 11.5%; χ² = 4.17, p = 0.025), and hospitalizations for heart failure (14.4% vs. 4.6%; χ² = 3.16, p = 0.026) were all significantly more frequent in the enoxaparin group. In contrast, stroke and revascularization rates remained low and comparable between groups. These findings suggest that fondaparinux not only reduced bleeding but was also associated with fewer ischemic complications over time.

**Table 3:** Ischemic complications among participants by treatment group (N=177) Data are presented as number (percentage). Statistical comparisons were performed using chi-square (χ²) tests for categorical variables and Fisher’s exact tests where appropriate. A p-value of <0.05 was considered statistically significant. c: statistical significance analyzed using chi-square test, f: statistical significance analyzed using Fisher's test; NA: not applicable

Ischemic complications	Fondaparinux (n=87)	Enoxaparin (n=90)	Test Statistics	p-value
	n(%)	n(%)		
In-hospital outcome				
Myocardial infarction	0(0.00%)	0(0.00%)	--	NA
Recurrent ischemia	8(9.20%)	16(17.78%)	χ²=2.10	0.095c
Stroke	0(0.00%)	0(0.00%)	--	NA
Heart failure	17(19.54%)	21(23.33%)	χ²=0.19	0.539c
Hospitalization for heart failure	0(0.00%)	0(0.00%)	--	NA
Revascularization	0(0.00%)	0(0.00%)	--	NA
At the 3-month follow-up				
Myocardial infarction	2(2.30%)	4(4.44%)	Fisher	0.682f
Recurrent ischemia	2(2.30%)	8(8.89%)	Fisher	0.099f
Stroke	1(1.15%)	3(3.33%)	Fisher	0.621f
Heart failure	7(8.05%)	14(15.56%)	χ²=1.72	0.109c
Hospitalization for heart failure	2(2.30%)	5(5.56%)	Fisher	0.444f
Revascularization	0(0.00%)	0(0.00%)	--	NA
At the 6-month follow-up				
Myocardial infarction	1(1.15%)	9(10.00%)	Fisher	0.008f
Recurrent ischemia	1(1.15%)	2(2.22%)	Fisher	0.613f
Stroke	0(0.00%)	1(1.11%)	Fisher	0.487f
Heart failure	13(14.94%)	21(23.33%)	χ²=1.50	0.084c
Hospitalization for heart failure	2(2.30%)	8(8.89%)	Fisher	0.052f
Revascularization	0(0.00%)	3(3.33%)	Fisher	0.115f
Total complications				
Myocardial infarction	3(3.45%)	13(14.44%)	Fisher	0.016f
Recurrent ischemia	10(11.49%)	22(24.44%)	χ²=4.17	0.025c
Stroke	1(1.15%)	4(4.44%)	Fisher	0.368f
Heart failure	25(28.74%)	32(35.56%)	χ²=0.66	0.332c
Hospitalization for heart failure	4(4.60%)	13(14.44%)	χ²=3.16	0.026c
Revascularization	0(0.00%)	3(3.33%)	Fisher	0.246f

Mortality and composite outcomes consistently trended higher in the enoxaparin group (Table 4). During index hospitalization, deaths occurred only in enoxaparin-treated patients (2.2%), though the difference was not statistically significant (p=0.497). By 3 months, mortality was higher in the enoxaparin group (12.2% vs. 9.2%), but again without statistical significance (χ²=0.17, p=0.515). At 6 months, mortality remained greater in the enoxaparin group (11.1% vs. 4.6%), with a borderline but non-significant difference (χ²=0.16, p=0.108). Cumulatively, total mortality was nearly twice as high in the enoxaparin group (25.6%) compared with the fondaparinux group (13.8%), and this difference approached statistical significance (χ²=3.15, p=0.050). Importantly, the composite outcome of death and major ischemic complications occurred significantly more frequently in enoxaparin patients (40.0% vs. 20.7%; χ²=6.90, p=0.005). These results underscore a clinically relevant advantage of fondaparinux in reducing the overall burden of adverse outcomes, particularly when both mortality and ischemic complications are considered together.

**Table 4:** Mortality and Composite End point among participants by groups (N=177) Data are presented as number (percentage). Statistical comparisons were performed using chi-square (χ²) tests for categorical variables and Fisher’s exact tests where appropriate. A p-value of <0.05 was considered statistically significant. c: statistical significance analyzed using chi-square test, f: statistical significance analyzed using Fisher's test

Timeline	Fondaparinux (n=87)	Enoxaparin (n=90)	Test Statistics	p-value
	N(%)	n(%)		
Mortality during index hospitalization	0(0.00%)	2(2.22%)	Fisher	0.497f
Mortality at 3-month follow-up	8(9.20%)	11(12.22%)	χ²=0.17	0.515c
Mortality at 6-month follow-up	4(4.60%)	10(11.11%)	χ²=0.16	0.108c
Mortality total	12(13.79%)	23(25.56%)	χ²=3.15	0.050c
Composite outcome	18(20.69%)	36(40.00%)	χ²=6.90	0.005c

In the adjusted logistic regression model, the treatment group emerged as the only independent predictor of the composite outcome (Table 5). Patients treated with fondaparinux had a significantly lower risk of experiencing the composite endpoint compared with those treated with enoxaparin (adjusted OR 0.336, 95% CI: 0.166-0.683, p=0.003). This indicates that fondaparinux use was associated with a nearly two-thirds reduction in the odds of composite adverse events after adjustment for baseline covariates. Other covariates, including age, sex, hypertension, diabetes, smoking, dyslipidemia, and time from symptom onset to intervention, did not show statistically significant associations with the composite outcome (all p>0.09). While dyslipidemia (OR 1.775, 95% CI: 0.837-3.765, p=0.135) and longer time to intervention (OR 1.115 per hour, 95% CI: 0.981-1.268, p=0.094) suggested trends toward higher odds of adverse events, these did not reach significance. Similarly, sex, diabetes, and hypertension demonstrated nonsignificant associations with lower odds, while smoking showed a nonsignificant increase in risk.

**Table 5:** Multivariable logistic regression analysis for composite outcome (N=177) Data are presented as odds ratios (OR) with 95% confidence intervals (CI). Statistical comparisons were performed using logistic regression analysis. A p-value of <0.05 was considered statistically significant.

Variable	B (Coefficient)	S.E.	Wald	df	p-value	Exp(B) (Adjusted OR)	95% CI (Lower – Upper)
Treatment group (fondaparinux vs. enoxaparin)	-1.089	0.361	9.104	1	0.003	0.336	0.166 – 0.683
Age (per year increase)	0.007	0.019	0.142	1	0.706	1.007	0.970 – 1.046
Sex (male vs. female)	-0.484	0.545	0.789	1	0.374	0.616	0.212 – 1.794
Hypertension (HTN: absent vs present)	-0.156	0.386	0.163	1	0.687	0.856	0.402 – 1.823
Diabetes mellitus (DM: absent vs present)	-0.303	0.363	0.698	1	0.404	0.739	0.363 – 1.503
Smoking (smoker vs. non-smoker)	0.264	0.519	0.259	1	0.611	1.302	0.471 – 3.601
Dyslipidemia (yes vs. no)	0.574	0.384	2.237	1	0.135	1.775	0.837 – 3.765
Time from onset to intervention	0.109	0.065	2.797	1	0.094	1.115	0.981 – 1.268

## Discussion

In this study, we compared the safety profile and ischemic outcomes of fondaparinux versus enoxaparin in NSTE-ACS patients in Bangladesh, with a particular focus on bleeding events, ischemic complications, mortality, and composite adverse outcomes. Our findings provide valuable insights into the real-world applicability of these anticoagulants in resource-limited settings and reinforce existing evidence from international and regional studies.

A major finding of this study was the significantly lower incidence of bleeding in patients treated with fondaparinux (6.90%) compared to enoxaparin (23.33%; p=0.002). Notably, baseline bleeding was significantly lower in fondaparinux patients (4.60%) versus enoxaparin (17.78%, p=0.006), while bleeding events remained low at 3-month (1.15% vs. 3.33%, p=0.747) and 6-month follow-ups (1.15% vs. 2.22%, p=0.970). Similarly, the OASIS-5 trial demonstrated a significant reduction in major in-hospital bleeding with fondaparinux compared to enoxaparin [15]. The Bundhun et al. meta-analysis, which included 7 studies and 9,618 patients, further confirmed that fondaparinux significantly reduces both minor (OR=0.40, p=0.00001) and major bleeding (OR=0.46, p=0.0001) compared to enoxaparin [16]. Collectively, these findings support fondaparinux as a superior anticoagulant for reducing bleeding risk, particularly in settings where blood transfusion resources may be limited.

In terms of ischemic complications, our study observed a significantly lower incidence of myocardial infarction (3.45% vs. 14.44%, p=0.016) and recurrent ischemia (11.49% vs. 24.44%, p=0.025) in fondaparinux-treated patients. Similarly, hospitalization due to heart failure was significantly lower (4.60% vs. 14.44%, p=0.026). The OASIS-5 study also reported that fondaparinux was non-inferior to enoxaparin in preventing ischemic outcomes while offering significant bleeding reduction [15]. Moreover, a multicenter registry study demonstrated a significant reduction in ischemic events with fondaparinux (subdistribution hazard ratio=0.59, p<0.05), further validating our findings [5]. These consistent results indicate that fondaparinux not only reduces bleeding risk but also effectively prevents ischemic complications in NSTE-ACS patients. While mortality rates were lower in fondaparinux-treated patients (13.79% vs. 25.56%, p=0.050, borderline significant), statistical significance was not reached. However, the composite endpoint (mortality + ischemic events) was significantly lower in the fondaparinux group (20.69%) compared to enoxaparin (40.00%, p=0.005). Similarly, the FAST-MI registry reported no significant difference in 1-year survival between fondaparinux and enoxaparin (94.6% vs. 91.7%), reinforcing our findings of a potential but non-significant mortality benefit [17]. However, the significant reduction in composite adverse events in our study is well-supported by both Almendro-Delia et al. (subdistribution hazard ratio=0.59, p<0.05) and OASIS-5 (HR=0.50, p=0.002), confirming fondaparinux's overall superiority in preventing major complications [5,15].

Our multivariable logistic regression analysis confirmed that fondaparinux significantly reduced the risk of composite adverse events (OR=0.336, 95% CI: 0.166-0.683, p=0.003), suggesting a 66.4% lower risk compared to enoxaparin. Importantly, other variables, including age (p=0.706), sex (p=0.374), hypertension (p=0.687), diabetes mellitus (p=0.404), smoking (p=0.611), and time to intervention (p=0.094), were not significant independent predictors of composite events. Additionally, the French Registry on Acute ST-elevation and non-ST-elevation Myocardial Infarction (FAST-MI) registry (2015) and Zhao et al. (2015) studies also found no significant associations between age, sex, or comorbidities and composite adverse outcomes (p>0.05) [17,18]. The meta-analysis by Bundhun et al. further corroborates fondaparinux's independent role in improving clinical outcomes [16]. The findings of this study have significant clinical relevance, particularly for resource-limited settings like Bangladesh, where access to blood transfusion and advanced cardiac care remains limited. Given the marked reduction in bleeding risk, fondaparinux could be a cost-effective and safer alternative to enoxaparin, particularly for patients at high risk of bleeding.

A cost-comparison study from Switzerland estimated that replacing enoxaparin with fondaparinux would generate healthcare savings of up to 63%, supporting its economic feasibility in resource-constrained environments [19]. Our study reinforces the superior safety and efficacy profile of fondaparinux over enoxaparin in NSTE-ACS patients, with significantly lower bleeding rates, fewer ischemic events, and a reduced composite endpoint risk. While mortality differences did not reach significance, the trend toward better survival outcomes with fondaparinux remains notable. Future studies with larger sample sizes and longer follow-up durations are warranted to confirm these findings, particularly in Bangladeshi and other South Asian populations.

Limitations of the study

The study was conducted in a single hospital with a small sample size. Therefore, the outcomes might not reflect the entire community.

## Conclusions

This prospective observational study demonstrated that fondaparinux is significantly superior to enoxaparin in reducing bleeding complications and ischemic events among NSTE-ACS patients in a Bangladeshi hospital setting. Fondaparinux-treated patients had markedly lower bleeding rates, fewer episodes of myocardial infarction and recurrent ischemia, and fewer hospitalizations due to heart failure, contributing to a significantly lower overall composite adverse event rate compared to those treated with enoxaparin. While total mortality differences showed a trend favoring fondaparinux, they did not reach statistical significance. Multivariable regression analysis confirmed fondaparinux as a strong independent predictor of improved clinical outcomes, with no significant effects observed from demographic or comorbid conditions. Given these findings, fondaparinux at a single daily dose of 2.5 mg presents a safer and potentially more effective anticoagulation strategy for managing NSTE-ACS patients, particularly in resource-limited healthcare environments such as Bangladesh. Future studies are warranted to further validate these results and assess the long-term clinical and economic implications of adopting fondaparinux broadly in similar settings.
